# Stability and Change in Genetic and Environmental Influences on Well-Being in Response to an Intervention

**DOI:** 10.1371/journal.pone.0155538

**Published:** 2016-05-26

**Authors:** Claire M. A. Haworth, S. Katherine Nelson, Kristin Layous, Kathryn Carter, Katherine Jacobs Bao, Sonja Lyubomirsky, Robert Plomin

**Affiliations:** 1 MRC Integrative Epidemiology Unit, School of Experimental Psychology & School of Social and Community Medicine, University of Bristol, Bristol, United Kingdom; 2 Department of Psychology, Sewanee: The University of the South, Sewanee, United States of America; 3 Department of Psychology, University of California Riverside, Riverside, United States of America; 4 Social, Genetic and Developmental Psychiatry Centre, King’s College London, London, United Kingdom; University of Hong Kong, HONG KONG

## Abstract

Genetic and environmental influences on complex traits can change in response to developmental and environmental contexts. Here we explore the impact of a positive activity intervention on the genetic and environmental influences on well-being and mental health in a sample of 750 adolescent twins. Twins completed a 10-week online well-being intervention, consisting of kindness and gratitude tasks and matched control activities. The results showed significant improvements both in well-being and in internalizing symptoms in response to the intervention activities. We used multivariate twin analyses of repeated measures, tracking stability and change in genetic and environmental influences, to assess the impact of this environmental intervention on these variance components. The heritability of well-being remained high both before and after the intervention, and the same genetic effects were important at each stage, even as well-being increased. The overall magnitude of environmental influences was also stable across the intervention; however, different non-shared environmental influences emerged during the intervention. Our study highlights the value of exploring the innovations in non-shared environmental influences that could provide clues to the mechanisms behind improvements in well-being. The findings also emphasize that even traits strongly influenced by genetics, like well-being, are subject to change in response to environmental interventions.

## Introduction

Twin analyses of subjective well-being have indicated significant heritability in the range of 30–50% [1]. DNA analyses with hundreds of thousands of participants are underway to identify some of the specific variants involved, but because of the very small effect size of each individual variant, hidden among the millions of common variations across the genome, these studies have not yet identified any robust associations [2]. Given the strong genetic influence on well-being, what are the implications for the design and effectiveness of interventions aimed at improving well-being? Because the importance of genetic and environmental influences can shift across development and in different environmental contexts [3–6], the magnitude and composition of these influences could plausibly change in response to an intervention.

The dynamic nature of genetic and environmental influences on a variety of outcomes has been demonstrated through observational studies [5]. For example, the heritability of intelligence [6] and body mass index (BMI) [7], among other traits, has been shown to increase with age. The heritability of cognitive and behavioural outcomes has also been shown to vary as a function of where people grow up [4]. Changes in genetic and environmental influences in response to historical shifts in environmental exposures have also been studied. For example, increases in the obesogenic environment, including increased access to high-calorie foods and sedentary occupations, have led to mean increases in weight, yet the genetic and environmental causes of individual differences have remained stable [8]. This pattern of results, however, is not always observed. For example, genetic influences on school performance increased dramatically with the introduction of formal education curricula after World War II [9]. Cohort (i.e., generational) differences in DNA associations are also now being uncovered (e.g., [10]. The empirical literature on the dynamic nature of genetic and environmental influences is growing, yet researchers still do not typically design their studies to truly investigate and understand how and why genetic and environmental influences might shift over time.

It is important to recognize that in terms of etiology, “what is” does not necessarily tell researchers about “what could be.” Genetic associations and estimates of the contribution of genetic and environmental variance components inform us about the etiological influences on the population as it is today, not what they could be if a new influence (such as an environmental intervention) is introduced. Constructs that show higher heritability are not necessarily more difficult to change; even a trait that is 100% heritable (as phenylketonuria [PKU] used to be, for example) could be modified with an appropriate intervention (e.g., diet). In the case of PKU, only by understanding the specific genetic and environmental causes and how they interacted were researchers able to develop an effective environmental intervention that targeted the disease’s mechanism [11].

Both laypeople who seek happiness and investigators who strive to increase it often assume that, because of its heritability, improving well-being is extremely difficult [12]. This assumption arises from the common misunderstanding that genetic influences on complex psychological constructs are deterministic and detrimental [13]. This misunderstanding is two-fold. First, the proportion of variance explained by genetic and environmental influences refers to population-level statistics, not to individual-level characteristics. When a trait is described as 50% heritable, this does not mean that 50% of an individual’s score on that trait is due to her genes and the rest is due to the environment. Rather, a heritability estimate indicates that, of the variation observed in a population, 50% of those differences between people are due to genetic differences between them. Second, genetic (and environmental) influences on complex constructs are not deterministic; proportions of variance represent probabilistic risk. It is possible to have genetic variants that confer risk for a particular outcome, but never show that outcome (e.g., genetic risk for heart disease), just as it is possible to experience risky or advantageous environments but not respond to them (e.g., good teaching does not always lead to good pupil performance).

Considering genetic and environmental influences on complex traits as dynamic factors has important implications for the science of behavior change and preventative medicine. As an initial step towards these aims, we developed a novel design that embeds a universal intervention within a twin study to assess the importance and stability of genetic and environmental influences on individual differences in response to an intervention. We applied this new method to interventions that have previously been shown to increase well-being [14].

### Interventions to Improve Well-Being

Improving well-being is a critical societal aim that has potential to spawn myriad positive downstream consequences. Well-being refers to positive aspects of a person’s mental health and is commonly conceptualized as encompassing subjective well-being (i.e., subjective ratings of life satisfaction and the experience of frequent positive and infrequent negative emotions [15]) and mental health (i.e., infrequent symptoms of anxiety and depression). Greater levels of well-being have been linked to various markers of success, including superior health, more positive social relationships, and improved workplace performance [16]. Notably, greater well-being precedes, as well as follows, these markers of successful outcomes [16], suggesting that improving well-being could directly or indirectly precipitate success in multiple life domains.

Growing evidence indicates that engaging in simple positive activities can reliably increase an individual’s well-being, and that these improvements are sustained at follow-ups from 1 to 6 months [17]. A meta-analysis of 51 positive activity interventions indicated significant improvements in well-being and significant attenuation of depressive symptoms [14,18].

Two key positive activities shown to increase happiness in randomized controlled interventions are performing acts of kindness and expressing gratitude [19,20]. Given that the efficacy of these interventions has been demonstrated, more attention is now being directed to understanding the moderators (e.g., individual difference characteristics) and mediators (e.g., positive thoughts) underlying intervention response [17]. Our study contributes to this research literature by investigating the role of genes and environments in creating these individual differences in intervention response.

### Genetics and Interventions

To date, most genetically sensitive interventions in the behavioral sciences have relied on candidate gene approaches (e.g., [21,22]). Genome-wide association studies (GWAS) offer a more robust and systematic method for identifying common genetic variants. However, the requirement of large discovery and replication samples make it logistically infeasible to combine genome-wide association discovery designs with intensive intervention programs at present. In light of these difficulties, the power of twin and family studies, which have more modest sample size requirements, and do not rely upon the a priori identification of specific genes (or environments), is a promising complementary method for exploring the overall pattern of genetic and environmental influence on intervention response [23].

Conducting interventions within twin and adoption studies provides a method for assessing both genetic and environmental influences on individual differences in intervention response [24]. A handful of quasi-experimental studies using different types of family designs have been published to date (see [25], for a review). In one example, the adoption design was used to assess the effectiveness of parenting strategies to reduce children’s behavioral problems [26]. Examples of observational twin designs, which consider changes in etiology in response to life transitions, include studies of well-being pre- and post-marriage [27], and the impact of the transition from primary to secondary school on school performance [28].

A novel application of the co-twin control design in educational research has recently been conducted [29]. The researchers used the quasi-experimental placement of twins in different classroom settings to investigate the causal relationship between teacher quality and reading outcomes. Using the co-twin control method rules out confounding from genetic and shared environmental sources, therefore allowing stronger causal interpretations about the effects of exposure. Such designs are particularly useful when it is difficult or unethical to experimentally manipulate exposure (e.g., to good and bad teacher quality). However, investigations in which it is possible to study the genetic and environmental response to interventions experimentally provide a more accurate indication of the specific intervention response, because the experimental design allows other factors to be more closely controlled. As described below, experimental studies also allow additional questions about genetic and environmental etiology to be addressed.

Few experimental gene-by-intervention interaction studies have been conducted in humans. One example is the acquisition of motor skills, which was investigated experimentally in a small study, finding that genetic influences explained more variance with increasing practice [30]. Furthermore, a growing literature has applied the experimental twin design to stress reactivity [31–33]. Such gene-by-stress interactions are equivalent to gene-by-intervention interactions that can be detected using experimental twin intervention studies. These stress reactivity studies highlight the benefits of multivariate twin analyses that can separate baseline and new genetic and environmental factors, rather than focusing on change scores that combine these etiological factors. We apply this multivariate twin design here to explore the continuity of baseline factors across the intervention, and to estimate the role of innovations in genetic and environmental influence specifically in response to the intervention.

### The Present Study

We aimed to assess whether using established methods for improving well-being could alter the pattern of genetic and environmental influence by embedding our universal intervention in a twin study. Two key questions are as follows: Will our environmental intervention increase the importance of environmental influences on well-being? And, what role do genes play in influencing the way in which people respond to the intervention? Specifically, we addressed the degree to which the same genetic and environmental influences are important before and after taking part in control activities and positive intervention activities. It is possible for the same genetic (or environmental) factors to explain more or less of the variance before and after the intervention, and it is also possible for new genetic (or environmental) influences to be introduced given our changing phenotype. Our multivariate twin design allows us to address both of these possibilities.

## Materials and Methods

### Sample

Participants in the Twins Well-Being Intervention Study (TWIST) were selected from the larger, population representative Twins Early Development Study (TEDS) [34]. Zygosity was assessed through a parent questionnaire of physical similarity [35]. Families were selected from TEDS to provide a subsample of same-sex twin pairs who were representative with respect to socioeconomic status, sex, and zygosity. Ethical approval for the study was provided by the Institute of Psychiatry research ethics committee at King’s College London (Ref: PNM/10/11-16).

After parents provided informed consent, twin participants logged in to our website to provide informed assent and begin the study. Of 885 twins who provided data at baseline, 807 (91.2%) were still actively involved at the follow-up assessment 9 weeks later. Twins who started but did not complete the study did not significantly differ in baseline well-being, mental health, or socioeconomic status from those who continued. Twins were rewarded a maximum £30 shopping voucher for completing the study, and families in which both twins completed all time points were also entered into a raffle for a pair of iPads. Twin pairs were excluded from the analyses if they had experienced birth complications (*n* = 24 individuals) or if both twins in the pair did not complete at least 4 of the 6 positive activities (*n* = 164 individuals). No significant differences in baseline well-being emerged between those who were excluded for not completing at least 4 activities and those who were included in the analyses.

The final sample included 750 individuals comprising 167 pairs of identical twins (59.8% female) and 208 pairs of non-identical same-sex twins (56.5% female). The mean age of the twins at the start of the study was 16.55 (*SD* = 0.51). The size of our sample was driven primarily by the power needed for twin analyses. Our sample of 167 pairs of identical twins and 208 pairs of non-identical twins provides 80% power to detect heritability of 0.40 at alpha = 0.05, which is within the range reported in the literature for the heritability of well-being measures. We performed our power calculation using the TwinPower tool: http://genepi.qimr.edu.au/cgi-bin/twinpower.cgi. Sample recruitment was reviewed weekly, and stopped once more than 400 families had agreed to take part in the study.

### Study Design

**Fig 1:**
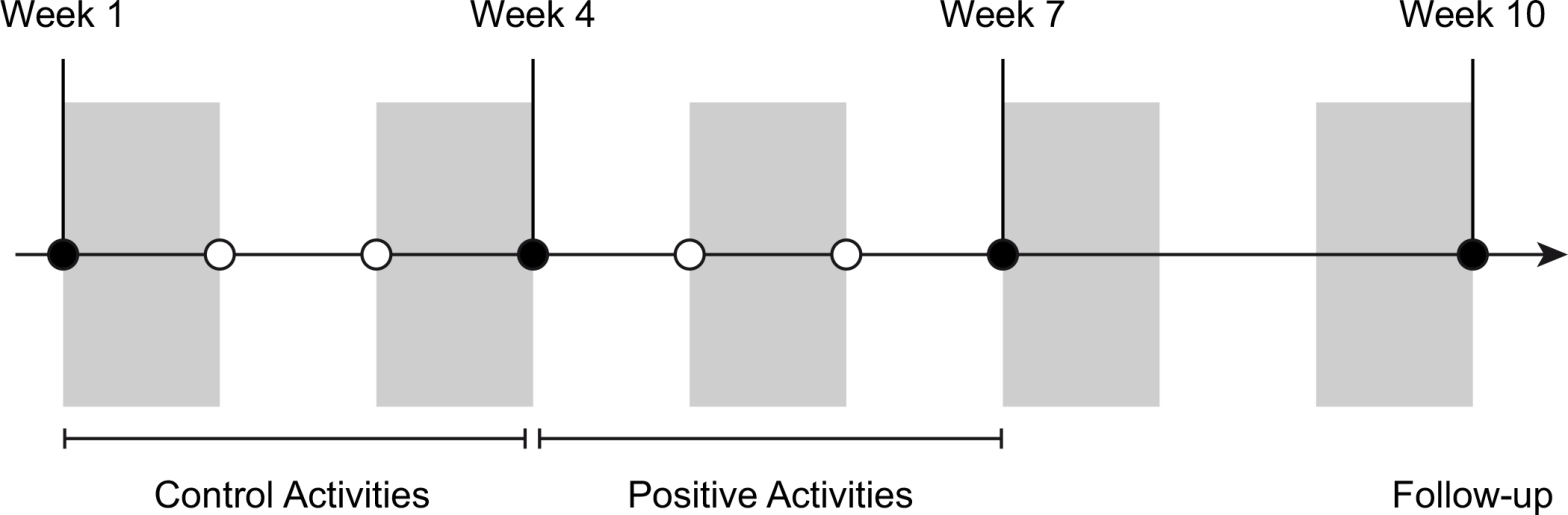
Study Design. Circles represent weeks when participants logged into the website to take part in the study. Filled circles reflect weeks during which participants completed scales measuring well-being and mental health.

All participants were informed that they would be engaging in a 10-week online study in which they would be instructed to perform activities to improve their well-being. Public knowledge of positive activity interventions makes it almost impossible to keep subjects blind to the intention of such a study. The inclusion of the control phase of our study (explained below) allows us to consider changes in means and etiology during the intervention phase of the study, beyond any potential impact of a placebo response. Twins participated in the study once per week for 6 weeks after baseline information was collected, as well as a final 3-week follow-up, yielding a total of 8 time points (see Fig 1). All twins completed two control activities each week during the first 3 weeks of the study and two positive activities each week during the second 3 weeks.

Our study design is a novel application of the twin method in intervention science, in which the use of within-individual control data allows us to maximally control for previous genetic and environmental influences on well-being, providing a strong test of any new genetic or environmental influences that are elicited specifically in response to the intervention activities. Other combinations of twins and intervention designs are possible, such as comparing two separate groups of twins who either did the control tasks or the intervention tasks, or by using a co-twin control method. We considered creating separate groups for the control tasks and the intervention tasks and using the data to conduct heterogeneity analyses to determine whether the proportion of genetic and environmental influence on well-being differed as a function of completing the intervention tasks. However, analyses to test differences in the size of genetic and environmental influence between groups require much larger sample sizes for adequate statistical power, potentially requiring thousands of pairs of twins per group, even for large effect sizes. Conducting an intensive intervention on a sample this size was prohibitively expensive. In addition, that design would only reveal whether the size of the genetic and environmental effects was the same or different (i.e., a quantitative difference) rather than revealing whether it is the same or different genetic and environmental effects active pre and post intervention (i.e., a qualitative difference), which a multivariate twin design allows.

We also ruled out a co-twin control design for this particular intervention, in a sample in which the twins still reside in the same household. A co-twin control design involves administering the intervention to just one member of a twin pair and treating their co-twin as the control. Because our intervention involves social interaction, it would be impossible to prevent a twin from discussing the intervention with their co-twin or from simply observing the intervention activities being performed by their co-twin. For these reasons, we opted to use a within-individual control design where everyone taking part in the study completes both the control activities and the intervention activities. This design allows us to examine both quantitative changes in genetic and environmental influence for the different stages of the study, but crucially also allows us to test for any qualitative differences in the genetic and environmental influences active throughout the study.

Activity Instructions. The two neutral control activities performed during the first 3 weeks (to visit three places and to describe one room in their home) were designed to parallel the two positive activities performed during the second 3 weeks (to perform three acts of kindness and to write a gratitude letter to an important individual in their lives). Participants were assigned their respective activities at the end of each week, to be performed on the following day. During subsequent weeks, they were first instructed to list their three kind acts or three places visited, and then instructed to spend 10 minutes writing a gratitude letter or describing a room in their house. Outcome variables were assessed at Week 1, Week 4, Week 7, and Week 10, which correspond to baseline, end of the control phase, end of the intervention phase, and the follow-up assessment. A detailed description of the tasks is provided in the supplementary materials (S1 Text).

### Measures

The analyses focus on our four main outcome variables to assess positive well-being and mental health. To assess well-being, we used the Subjective Happiness Scale (SHS) [36] and the Brief Multidimensional Student Life Satisfaction Scale (BMSLSS) [37]. The SHS consists of four items (three positively worded, and one negatively worded) rated on a 7-point scale (e.g., “Some people are generally very happy. They enjoy life regardless of what is going on, getting the most out of everything. To what extent does this describe you?” 1 = *not at all*, 7 = *a great deal*). The BMSLSS consists of six items assessing satisfaction with family, friends, school experience, self, where you live, and overall satisfaction with life, rated on a scale ranging from 1 (*very dissatisfied*) to 7 (*very satisfied*). Both measures demonstrated good internal consistency reliability, with Cronbach’s αs ranging from .88 to .89 for the SHS and .86 to .89 for the BMSLSS across all time points.

To assess mental health, we used the short Mood and Feelings Questionnaire [38] to measure symptoms of depression and the State-Trait Anxiety Inventory [39] to measure anxiety. The Moods and Feelings questionnaire consists of 13 items rated on a 3-point scale (*not true*, *quite true*, and *very true*). The twins rated how true each statement was for them over the past week (e.g., “I didn’t enjoy anything at all”). The short State-Trait Anxiety Inventory consists of 6 items rated on a 4-point scale (*not at all*, *somewhat*, *moderately so*, and *very much so*). The twins reported how they felt right now in response to each statement (e.g., “I am worried”). Both measures demonstrated good internal consistency reliability, with Cronbach’s αs of .90 for depression and .80 to .84 for anxiety across all time points of the study.

We calculated the scores for each measure by taking the mean of the items (requiring at least 50% of the items to be non-missing) and reversed the scoring where necessary so that higher values denote greater well-being or fewer symptoms of depression and anxiety. These scores for each time point were then standardized on baseline and combined into two composites indexing well-being (happiness and life satisfaction) and mental health (depression and anxiety) at each time point.

### Analyses of Phenotypic Intervention Response

Overall changes in well-being and mental health were assessed using multilevel growth curve modeling to account for repeated measurements nested within individuals, as well as individuals nested within twin pairs. We compared the fit of an unconditional growth model and a piecewise linear growth model. The piecewise linear growth model allowed us to assess changes in well-being and mental health associated with the control period and the positive activity and follow-up period [40]. In both models, γ_10_ is the estimate of linear slope across the entire study. In Model 2, γ_20_ reflects the additional changes in slope beginning with the intervention period. In both models, the intercept, and both estimates of slope (Time and Time 2) were free to vary.

### Analyses of Genetic and Environmental Stability and Change

To prepare our data for twin analyses, a van der Waerden rank transformation was applied to all measures to correct for negative skew. In addition, as is standard in twin analyses, all measures were corrected for the mean effects of age and sex using a regression procedure [41].

Twin analyses allow the estimation of the relative contributions of genes and environments to individual differences in measured traits [42]. Twin intraclass correlations were calculated, providing an initial indication of additive genetic (A), shared environmental (C), and non-shared environmental (E) factors. Additive genetic influence, commonly known as heritability, is estimated as twice the difference between the identical and non-identical twin correlations. The contribution of the shared environment, which makes members of a family similar, is estimated as the difference between the identical twin correlation and heritability. Non-shared environments, (environments specific to individuals), are estimated by the difference between the identical twin correlation and 1, because they are the only source of variance making identical twins different. Estimates of the non-shared environment also include measurement error.

Structural equation model-fitting allows more complex analyses, formal tests of significance, and the calculation of confidence intervals [43]. A Cholesky decomposition was fitted to the data using Mx [44]. The Cholesky decomposition allows the estimation of continuity and change in the genetic and environmental parameters across the four stages of the study: baseline, control, intervention, and follow-up. The first genetic factor (A1) represents genetic influences on baseline. The extent to which these same genes also influence the outcome at control, intervention and follow-up is also estimated. The second genetic factor (A2) represents genetic influences on the control stage that are independent of those influencing baseline. The extent to which these genes also influence the outcome at intervention and follow-up is also estimated. The third genetic factor (A3) indexes genetic influences on the intervention stage that are independent of genetic influences shared with baseline and control. That is, these genetic influences are specifically elicited by the intervention activities. The impact of these genes on follow-up is also estimated. Finally, the fourth genetic factor (A4) represents residual genetic influences on the outcome at follow-up. The same decomposition is done for the shared environmental and non-shared environmental influences (C1-4 and E1-4, respectively).

## Results

### Phenotypic Changes in Well-Being and Mental Health

**Fig 2:**
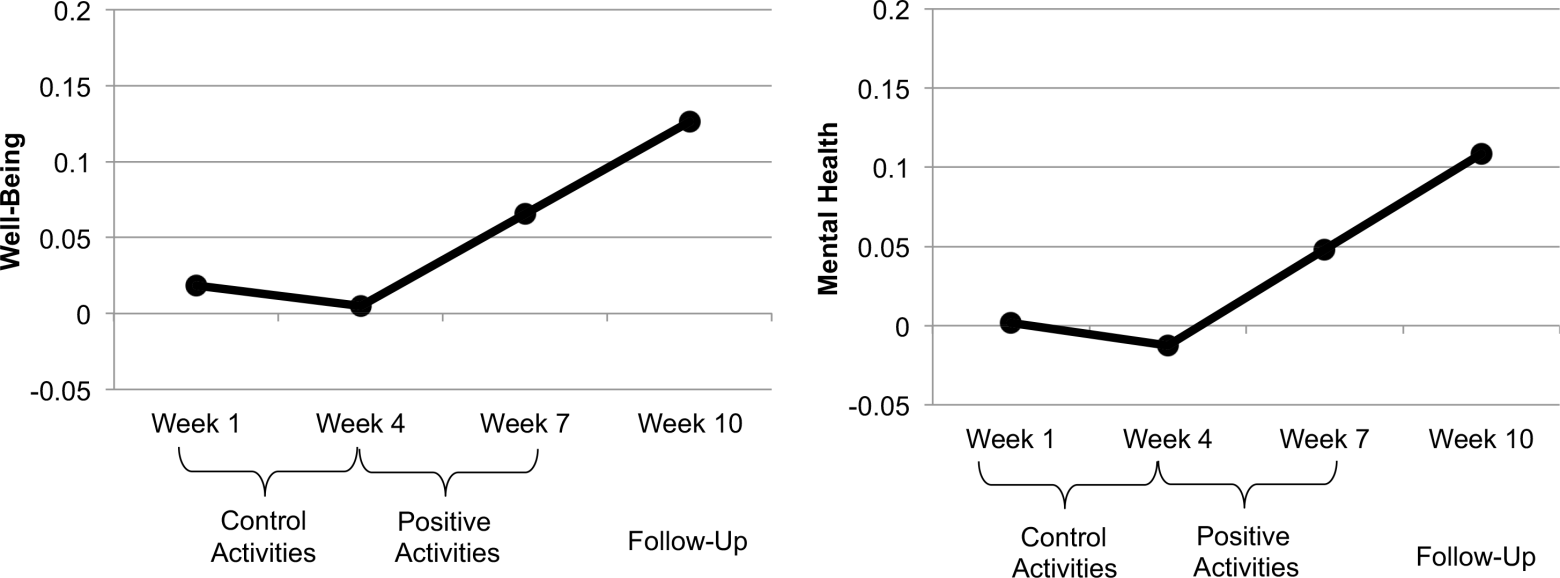
Model-Predicted Changes in Well-Being and Mental Health. These figures represent the model-predicted changes in well-being and mental health associated with the control period, the intervention period, and the follow-up period, which were estimated from our final piecewise growth models. Well-Being = Subjective Happiness Scale, Brief Multidimensional Student’s Life Satisfaction Scale; Mental Health = State-Trait Anxiety Inventory, Mood and Feelings Questionnaire (reversed so higher scores indicate fewer symptoms of depression and anxiety). Model fit-statistics are shown in S3 Table.

Supplementary Tables (S1 Table and S2 Table) show the means and standard deviations for the well-being and mental health measures at each stage of the study and the phenotypic correlations between these measures. Multilevel growth curve models were used to assess changes in well-being and mental health in response to the intervention. A piecewise model, with two time variables provided a better fit to the data than the unconditional growth model (Fig 2, and S3 Table). The results indicated that the twins did not demonstrate any significant changes in well-being or mental health during the control period, γ_10_s = 0.01, *ps* > .45, but they showed improvements in both well-being, γ_20_ = 0.07, S.E. = 0.02, *t*(2195) = 3.23, *p* = .001, and mental health, γ_20_ = 0.07, S.E. = 0.03, *t*(2195) = 2.17, *p* = .03, after practicing gratitude and kindness. These improvements in well-being and mental health continued through the 3-week follow-up (see Fig 2 and S3 Table). The small and non-significant fluctuations during the control phase are likely due to measurement error or a weak placebo effect. That these changes are not significant gives us confidence that the increase in well-being was in fact due to the positive activities, as when these start, we observe a significant change in the slope in our multilevel model.

### Changes in Etiology in Response to the Intervention

Twin analyses addressed the genetic and environmental origins of individual differences rather than group means. One question is whether heritability changes with intervention. Across both well-being and mental health, identical twin correlations were greater than the non-identical twin correlations, indicating genetic influence on individual differences in both constructs at all four stages (baseline, control, intervention, and follow-up; see Table 1).

**Table 1:** Intraclass Correlations for Identical and Non-Identical Twin Pairs.

Measure	Zygosity	Baseline (Week 1)	Control (Week 4)	Intervention (Week 7)	Follow-Up (Week 10)
Well-Being	Identical	0.55 (*N* = 165)	0.55 (*N* = 160)	0.50 (*N* = 155)	0.53 (*N* = 164)
	Non-Identical	0.32 (*N* = 206)	0.21 (*N* = 200)	0.25 (*N* = 198)	0.20 (*N* = 199)
Mental Health	Identical	0.34 (*N* = 165)	0.40 (*N* = 160)	0.40 (*N* = 155)	0.37 (*N* = 164)
	Non-Identical	0.27 (*N* = 206)	0.13 (*N* = 200)	0.08^ (*N* = 198)	0.12 (*N* = 199)

*Note*. *N* = complete twin pairs. All correlations are significant at *p* < .05 with one exception marked ^ where *p* = .123.

Twin analyses provided estimates of the importance of genes and environments at each stage (see A_total_, C_total_, and E_total_ in Tables 2 and 3). Results indicate that genetic influences explained 48% of the variance in well-being at baseline. Furthermore, the genetic influences remained consistent across the three subsequent stages of the study: 49% at the control stage, 45% at the intervention stage, and 48% at follow-up. Similarly stable results were found for mental health (24%, 35%, 35%, and 28% of the variance, respectively). The small fluctuations in heritability are not significant, as indicated by the overlapping confidence intervals (Tables 2 and 3). Results for shared and non-shared environmental influences also indicate nonsignificant changes in the magnitude of the effect across the study. However, even in the absence of quantitative changes in the magnitude of genetic and environmental influence, it is possible for qualitatively different environmental influences or genetic factors to emerge at the different stages of the study. Given that the intervention activities (e.g., doing acts of kindness for others) could be creating new environmental experiences, we might expect to find new environmental factors in response to these tasks.

**Table 2:** Parameter Estimates and 95% Confidence Intervals of Genetic and Environmental Influence from Multivariate Twin Analyses for Well-Being.

	Baseline (Week 1)	Control (Week 4)	Intervention (Week 7)	Follow-Up (Week 10)
A1	0.48 (.20-.64)	0.48 (.24-.62)	0.41 (.16-.58)	0.44 (.21-.58)
A2		0.02 (.00-.05)	0.03 (.00-.08)	0.03 (.00-.10)
A3			0.01 (.00-.03)	0.01 (.00-.04)
A4				0.00 (.00-.04)
A_total_	0.48 (.20-.64)	0.49 (.26-.63)	0.45 (.19-.60)	0.48 (.26-.60)
C1	0.07 (.00-.30)	0.05 (.00-.24)	0.06 (.00-.26)	0.03 (.00-.20)
C2		0.00 (.00-.02)	0.00 (.00-.04)	0.00 (.00-.04)
C3			0.00 (.00-.02)	0.00 (.00-.02)
C4				0.00 (.00-.02)
C_total_	0.07 (.00-.30)	0.05 (.00-.24)	0.06 (.00-.26)	0.03 (.00-.20)
E1	0.44 (.36-.55)	0.23 (.16-.32)	0.22 (.15-.32)	0.21 (.14-.30)
E2		0.22 (.18-.26)	0.06 (.04-.10)	0.07 (.04-.10)
E3			0.20 (.17-.24)	0.04 (.03-.07)
E4				0.18 (.14-.21)
E_total_	0.44 (.36-.55)	0.45 (.36-.56)	0.49 (.39-.60)	0.49 (.40-.60)

*Note*. A_total_ = total additive genetic influence on each measure; C_total_ = total shared environmental influence on each measure; E_total_ = total non-shared environmental influence on each measure. A1/C1/E1 = genetic/shared environmental/non-shared environmental influence on first measure and its influence on the remaining measures. A2/C2/E2 = genetic/shared environmental/non-shared environmental influence on second measure (independent of influences shared with first measure) and its influence on the remaining measures. A3/C3/E3 = genetic/shared environmental/non-shared environmental influence on third measure (independent of influences shared with first and second measures) and its influence on the remaining measure. A4/C4/E4 = genetic/shared environmental/non-shared environmental influence specific to last measure. The total estimate for A, C or E may differ slightly from the sum of the A/C/E1-4 estimates due to rounding of the estimates to two decimal places.

**Table 3:** Parameter Estimates and 95% Confidence Intervals of Genetic and Environmental Influence from Multivariate Twin Analyses for Mental Health.

	Baseline (Week 1)	Control (Week 4)	Intervention (Week 7)	Follow-Up (Week 10)
A1	0.24 (.04-.44)	0.26 (.04-.48)	0.34 (.10-.48)	0.27 (.04-.44)
A2		0.09 (.00-.17)	0.01 (.00-.14)	0.01 (.00-.15)
A3			0.01 (.00-.09)	0.00 (.00-.13)
A4				0.00 (.00-.13)
A_total_	0.24 (.04-.44)	0.35 (.12-.49)	0.35 (.15-.48)	0.28 (.06-.45)
C1	0.13 (.00-.31)	0.05 (.00-.21)	0.01 (.00-.14)	0.01 (.00-.13)
C2		0.00 (.00-.11)	0.00 (.00-.08)	0.03 (.00-.14)
C3			0.00 (.00-.07)	0.02 (.00-.12)
C4				0.00 (.00-.12)
C_total_	0.13 (.00-.31)	0.05 (.00-.22)	0.02 (.00-.16)	0.05 (.00-.23)
E1	0.63 (.52-.74)	0.20 (.12-.29)	0.16 (.09-.24)	0.17 (.10-.26)
E2		0.41 (.34-.49)	0.09 (.05-.15)	0.08 (.04-.13)
E3			0.38 (.31-.45)	0.04 (.02-.08)
E4				0.38 (.31-.45)
E_total_	0.63 (.52-.74)	0.61 (.50-.73)	0.63 (.51-.75)	0.67 (.55-.79)

*Note*. A_total_ = total additive genetic influence on each measure; C_total_ = total shared environmental influence on each measure; E_total_ = total non-shared environmental influence on each measure. A1/C1/E1 = genetic/shared environmental/non-shared environmental influence on first measure and its influence on the remaining measures. A2/C2/E2 = genetic/shared environmental/non-shared environmental influence on second measure (independent of influences shared with first measure) and its influence on the remaining measures. A3/C3/E3 = genetic/shared environmental/non-shared environmental influence on third measure (independent of influences shared with first and second measures) and its influence on the remaining measure. A4/C4/E4 = genetic/shared environmental/non-shared environmental influence specific to last measure. The total estimate for A, C or E may differ slightly from the sum of the A/C/E1-4 estimates due to rounding of the estimates to two decimal places.

**Fig 3:**
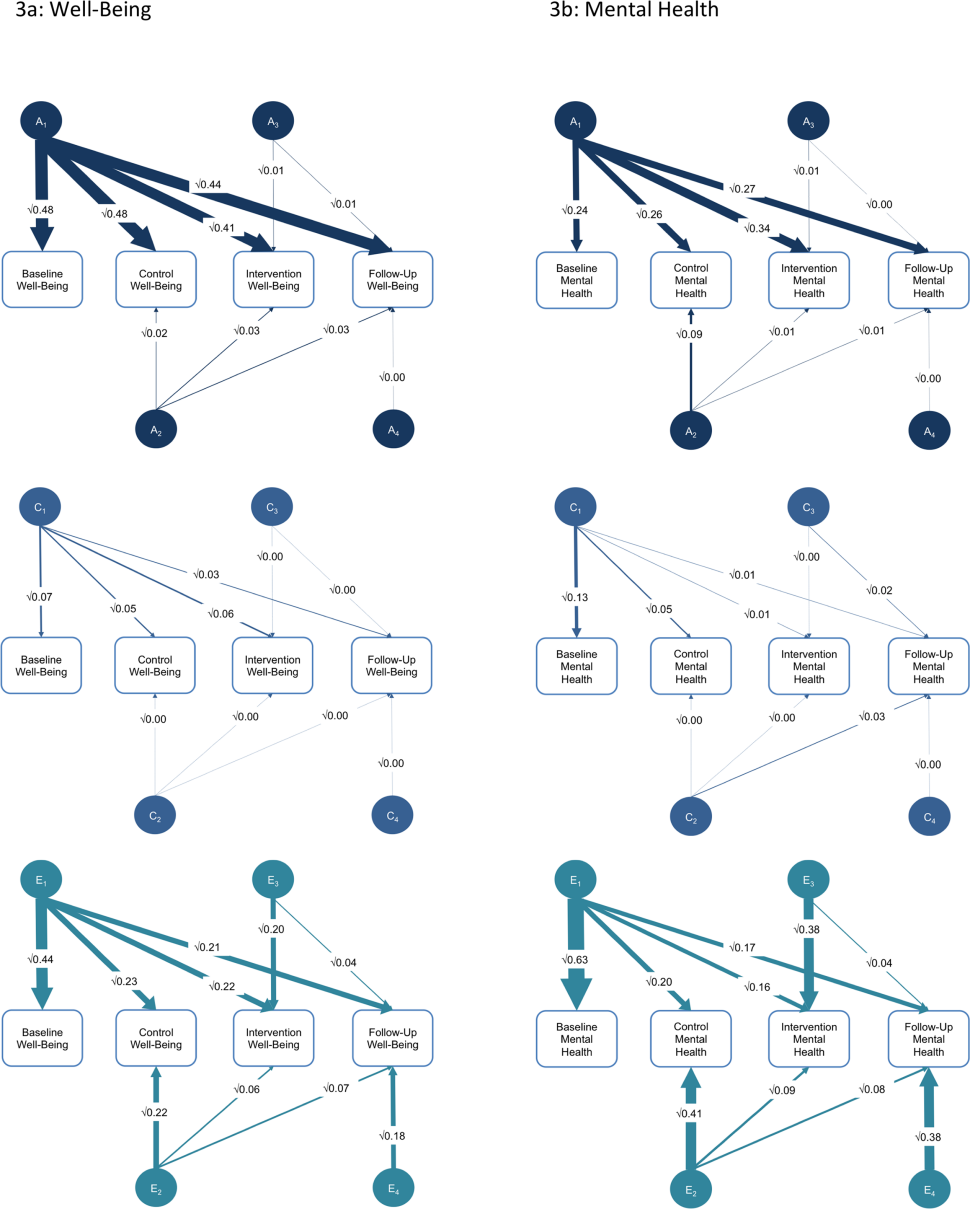
Twin Analyses of Continuity and Change in Genetic and Environmental Influence Across the Intervention (3a: Well-Being; 3b: Mental Health). These figures represent the standardized results from the multivariate twin model-fitting analyses using a Cholesky decomposition. This is a hierarchical analysis that highlights continuity and change across the study period. Line weights and intensities are used to represent the size of the parameter estimates. Confidence intervals for these parameters are shown in Tables 2 and 3. A = additive genetic; C = shared environment; E = non-shared environment. A1/C1/E1 = genetic/shared environmental/non-shared environmental influence on baseline and its influence on the remaining measures. A2/C2/E2 = genetic/shared environmental/non-shared environmental influence on control phase (independent of influences shared with baseline) and its influence on the remaining measures. A3/C3/E3 = genetic/shared environmental/non-shared environmental influence on intervention (independent of influences shared with baseline and control phase) and its influence on the remaining measure. A4/C4/E4 = genetic/shared environmental/non-shared environmental influence specific to follow-up.

Our multivariate twin analyses for well-being and mental health indicate that genetic influences at baseline can account for genetic influences at the later stages of the study (see Fig 3 and Tables 2 and 3). The estimates for the importance of the shared environment (environments that make family members more similar) are very small, but the baseline variance again accounts for almost all of the shared environmental influences across the different stages of the intervention. Only the non-shared environmental influences (environments that are unique to individuals) show innovations across the study. We find new non-shared environmental influences at every stage of the study (the E2, E3, and E4 factors in Fig 3), as well as some non-shared environmental influences that contribute to continuity across the study (E1 factor). For well-being, 42% [.20/ (.22 + .06 + .20) = .42] of the non-shared environmental influence on the intervention stage of the study is specific to that stage. For mental health, the equivalent statistic is 60% [.38/(.16 + .09 + .38) = .60]. Although the overall magnitude of non-shared environmental influence remains the same, there are qualitative differences in the environmental experiences that matter at each stage of the study.

## Discussion

Our twin analyses revealed minimal changes in the overall magnitude of genetic and environmental influence on individual differences during the intervention, despite significant improvements in overall well-being. Our novel design allowed us to show that the genetic factors important for intervention response were the same as those influencing baseline well-being scores.

Changes in well-being across the study were due to new environmental influences. These environmental influences are of the non-shared variety, meaning they are unique to individuals within a twin pair, contributing to differences in their outcomes. These non-shared experiences may have been, for example, interacting with different people while doing their acts of kindness, or in simply experiencing or interpreting the intervention activities differently from their co-twin.

Our results are similar to those for BMI, in which historical changes in the obesogenic environment have led to mean increases in weight, yet the causes of individual differences have remained stable [8]. For both BMI and our well-being intervention, genetic influences are a critical and stable influence on the variance in the population before and after the change in the environment. Yet it is the change in the environment that led to a shift in the population distribution.

Our results underscore the notion that finding significant heritability is not a barrier to effective interventions. The magnitude of heritability does not necessarily reveal anything about whether it will be possible to change a trait. Likewise, the relatively low heritability of many traits in childhood compared to adulthood should not be used as the primary rationale for early intervention programs. Instead, investigators should be taking advantage of the dynamic nature of genetic and environmental influences by using interventions to build on people’s strengths and overcome their weaknesses.

Advancing knowledge of the baseline influences on well-being is one step towards understanding individual differences, but more experimental investigations of gene-environment interplay are also needed. Identifying which specific environmental experiences and which specific variations in people’s DNA are involved is another crucial step towards designing better interventions that target the mechanisms of behavior change. If our finding that baseline genetic influences also influence the way people respond to positive activity interventions is replicated, then it may not be necessary to combine intensive intervention designs with expensive genome-wide discovery methods to identify variants for intervention response. Instead, DNA variants identified via traditional genome-wide investigations could be used to explore intervention response, for example, by using a recall-by-genotype method to select samples for specific interventions.

### Limitations and Future Directions

In line with other public health (universal) interventions, we found small mean effect sizes for the intervention boost in well-being and mental health. As a result, we cannot rule out the possibility that larger mean intervention effects could yield changes in the genetic and environmental origins of individual differences. In addition, although innovations in non-shared environmental influences explained changes in well-being across the study, estimates of non-shared environment include measurement error. However, given that the same measures and methods were used throughout the study, we would expect measurement error to be mostly correlated across the different assessments, and therefore captured in the baseline estimate for non-shared environmental influence. About half of the non-shared environmental influences at baseline showed a stable effect on the outcome throughout our intervention, partly reflecting shared method variance and error. New non-shared environmental influences in response to the intervention that were completely uncorrelated with the influences at previous assessments are therefore most likely genuine environmental experiences. An important future direction for our work will be in identifying which specific environmental experiences explain our non-shared environmental variance. In addition, including measurements from multiple informants and developing more objective measurements of well-being would allow a more stringent test of true environmental variance unconfounded by measurement error.

As discussed earlier, alternate twin and family studies could be used to combine genetically sensitive approaches with interventions. We chose a within-individual control design that allowed us to conduct multivariate analyses to investigate both qualitative and quantitative changes in etiology across the intervention. Future investigators could consider using a co-twin control design, which might be more effective with older twins who are not living in the same home. Another innovation to the design would be increasing the number of measurement occasions across the study, which would allow estimation of genetic and environmental influences on both the slope and intercept of well-being across the different stages of the study.

Additionally, we cannot determine whether the intervention effects were driven by the acts of kindness task or the gratitude letter task, or whether both were important. Both positive activities have been shown to significantly boost happiness and other favorable outcomes in previous work (see [45] for a review), but there are likely to be individual differences in preferences for these activities as well. Our aim here was to generate an improvement in well-being and to track changes in genetic and environmental influence, rather than to distinguish the effectiveness of the different tasks. Future studies could consider the fit of the positive activity to an individual’s personal characteristics.

Finally, our intervention was only a short-term study lasting 10 weeks. As a result, we are neither able to establish whether the intervention has lasting effects on well-being, nor whether delayed changes to genetic and environmental influence might emerge in the long-term. We also cannot establish whether our results are specific to a well-being intervention with teenagers. The adolescent years mark an important transition in terms of increases in mental health problems and decreases in overall well-being [46,47]. A significant future direction for this research will be to consider developmental specificity of the intervention effects across the lifespan, as well as the effects of genetic and environmental influences on our ability to change our well-being at different ages. Previous genetically informative research on the well-being of adolescents has suggested a similar pattern of etiology to adulthood well-being and mental health [48], and work on positive activity interventions has so far indicated that tasks such as acts of kindness and writing letters of gratitude are effective with different age groups [19,49]. One intriguing future direction will be to investigate whether positive activity interventions in childhood and adolescence help make young people more resilient as they grow up.

### Conclusions

Our findings show that genetic influences on well-being pre-intervention are largely the same genetic influences that are important in explaining individual differences in response to the intervention. Notably, new environmental influences that do not contribute to familial similarity did explain changes in well-being in response to the intervention. Understanding what specific experiences account for this new non-shared environmental variance will help elucidate the mechanisms that lead to improvements in well-being. The importance of baseline characteristics, including stable genetic factors, could provide clues to how to increase the effectiveness of positive activity interventions by improving our understanding of the fit between an intervention and the individual [17]. Rather than being a barrier to the pursuit of happiness, evidence of heritability yields clues to its success.

## Supporting Information

S1 Table Standardized Means (Standard Deviations) for Well-Being and Mental Health. (DOCX)

S2 Table Phenotypic Correlations Within Well-Being and Mental Health Across Time. (DOCX)

S3 Table Fit Statistics for Multilevel Models of Intervention Response. (DOCX)

S1 Text Intervention instructions: The text used in the intervention for both the control activities and the positive activities. (DOCX)
