# Supplementary material for: Stability and Change in Genetic and Environmental Influences on Well-Being in Response to an Intervention
Source: PLoS One. 2016 May 26;11(5):e0155538. doi: 10.1371/journal.pone.0155538 (PMC4881940; doi:10.1371/journal.pone.0155538)
Supplement: S1 Table — (DOCX) [file pone.0155538.s001.docx]

**Supplementary Table 1: Standardized Means (Standard Deviations) for Well-Being and Mental Health**

|  | Baseline  (Week 1) | | Control  (Week 4) | | Intervention  (Week 7) | | Follow-Up  (Week 10) | |
| --- | --- | --- | --- | --- | --- | --- | --- | --- |
|  | *M (SD)* | *N* | *M (SD)* | *N* | *M (SD)* | *N* | *M (SD)* | *N* |
| Well-Being | 0.03 (0.92) | 374 | 0.04 (0.94) | 370 | 0.13 (0.94) | 364 | 0.11 (0.93) | 370 |
| Mental Health | -0.02 (0.92) | 374 | -0.02 (0.94) | 370 | 0.11 (0.89) | 364 | 0.11 (0.85) | 370 |

*Note*. Well-Being = Subjective Happiness Scale, Brief Multidimensional Student’s Life Satisfaction Scale; Mental Health= State-Trait Anxiety Inventory, Mood and Feelings Questionnaire. *N* = one randomly selected member of each twin pair. Scores are standardized on baseline measures for the whole sample.
