# Supplementary material for: Stability and Change in Genetic and Environmental Influences on Well-Being in Response to an Intervention
Source: PLoS One. 2016 May 26;11(5):e0155538. doi: 10.1371/journal.pone.0155538 (PMC4881940; doi:10.1371/journal.pone.0155538)
Supplement: S2 Table — (DOCX) [file pone.0155538.s002.docx]

**Supplementary Table 2: Phenotypic Correlations Within Well-Being and Mental Health Across Time**

|  | Well-Being | Baseline  (Week 1) | Control  (Week 4) | Intervention  (Week 7) | Follow-Up  (Week 10) |
| --- | --- | --- | --- | --- | --- |
| Mental Health |  |  |  |  |  |
| Baseline  (Week 1) | |  | .86  (*N* = 368) | .82  (*N* = 362) | .81  (*N* = 368) |
| Control  (Week 4) | | .68  (*N* =368) |  | .86  (*N* = 359) | .86  (*N* = 364) |
| Intervention  (Week 7) | | .61  (*N* = 362) | .73  (*N* = 359) |  | .89  (*N* = 361) |
| Follow-Up  (Week 10) | | .61  (*N* = 368) | .72  (*N* = 364) | .71  (*N* = 361) |  |

*Note*. Correlations for well-being are shown above the diagonal, and correlations for mental health are shown below the diagonal. *N* = one randomly selected member of each twin pair. All correlations are significant at *p* < .01. Correlations performed on rank-transformed variables.
