# Supplementary material for: Stability and Change in Genetic and Environmental Influences on Well-Being in Response to an Intervention
Source: PLoS One. 2016 May 26;11(5):e0155538. doi: 10.1371/journal.pone.0155538 (PMC4881940; doi:10.1371/journal.pone.0155538)
Supplement: S3 Table — (DOCX) [file pone.0155538.s003.docx]

**Supplementary Table 3: Fit Statistics for Multilevel Models of Intervention Response**

Table S3a

Model Parameters (Standard Errors) and Goodness-of-Fit for Changes in Well-Being Through Follow-Up

|  | *Effect* | *Parameter* | *Model 1:*  *Unconditional Growth Model* | *Model 2:*  *Piecewise Model* |
| --- | --- | --- | --- | --- |
| ***Fixed Effects*** |  |  |  |  |
| Status at Baseline, π_oi_ | Intercept | γ_00_ | -0.003 (0.04) | 0.02 (0.04) |
| Rate of Change, π_1i_ | Time | γ_10_ | .04*** (0.007) | -0.01 (0.02) |
|  | Time 2 | γ_20_ |  | 0.07** (0.02) |
| ***Random Effects*** |  |  |  |  |
| Level 1 | Residual | σ^2^_ε_ | 0.11 | 0.11 |
| Level 2 | Intercept | σ^2^_0_ | 0.42 | 0.39 |
|  | Time | σ^2^_1_ | 0.01 | 0.03 |
|  | Time 2 | σ^2^_2_ |  | 0.01 |
| Level 3 | Intercept | σ^2^_0_ | 0.32 | 0.3 |
|  | Time | σ^2^_1_ | 0.002 | 0.01 |
|  | Time 2 | σ^2^_2_ |  | 0.01 |
| ***Goodness-of-fit*** |  |  |  |  |
|  | Deviance |  | 4617.99 | 4579.44 |
|  | AIC |  | 4635.99 | 4611.44 |
|  | BIC |  | 4689.9 | 4707.27 |

**p* < .05. ***p* < .01. ****p* < .001.

*Note*. In both models, the intercept parameter estimate (γ_00_) represents the average well-being score at baseline across the sample. In both models, γ_10_ is the estimate of linear slope across the entire study. In Model 2, γ_20_ reflects the additional changes in slope beginning with the intervention period. In both models, the intercept, and both estimates of slope (Time and Time 2) were free to vary. In all models, repeated measures were nested within individuals, and individuals were nested within twin pairs.

Table S3b

Model Parameters (Standard Errors) and Goodness-of-Fit for Changes in Mental Health Through Follow-Up

|  | *Effect* | *Parameter* |  | *Model 1:*  *Unconditional Growth Model* | *Model 2:*  *Piecewise Model* |
| --- | --- | --- | --- | --- | --- |
| ***Fixed Effects*** |  |  |  |  |  |
| Status at Baseline, π_oi_ | Intercept | γ_00_ |  | -0.02 (0.04) | 0.001 (0.04) |
| Rate of Change, π_1i_ | Time | γ_10_ |  | 0.04*** (0.01) | -0.01 (0.03) |
|  | Time 2 | γ_20_ |  |  | 0.07* (0.03) |
| ***Random Effects*** |  |  |  |  |  |
| Level 1 | Residual | σ^2^_ε_ |  | 0.24 | 0.23 |
| Level 2 | Intercept | σ^2^_0_ |  | 0.36 | 0.33 |
|  | Time | σ^2^_1_ |  | 0.001 | 0.02 |
|  | Time 2 | σ^2^_2_ |  |  | 0.03 |
| Level 3 | Intercept | σ^2^_0_ |  | 0.23 | 0.22 |
|  | Time | σ^2^_1_ |  | 0.01 | 0.04 |
|  | Time 2 | σ^2^_2_ |  |  | 0.04 |
| ***Goodness-of-fit*** |  |  |  |  |  |
|  | Deviance |  |  | 6016.8 | 5993.07 |
|  | AIC |  |  | 6034.8 | 6025.07 |
|  | BIC |  |  | 6088.7 | 6120.9 |

**p* < .05. ***p* < .01. ****p* < .001.

*Note.* In both models, the intercept parameter estimate (γ_00_) represents the average mental health score at baseline across the sample. In both models, γ_10_ is the estimate of linear slope across the entire study. In Model 2, γ_20_ reflects the additional changes in slope beginning with the intervention period. In both models, the intercept, and both estimates of slope (Time and Time 2) were free to vary. In all models, repeated measures were nested within individuals, and individuals were nested within twin pairs.
