# Supplementary material for: Stability and Change in Genetic and Environmental Influences on Well-Being in Response to an Intervention
Source: PLoS One. 2016 May 26;11(5):e0155538. doi: 10.1371/journal.pone.0155538 (PMC4881940; doi:10.1371/journal.pone.0155538)
Supplement: S1 Text — (DOCX) [file pone.0155538.s004.docx]

**Intervention Instructions**

| Activity | Instructions |
| --- | --- |
| Control Activity: Three Locations | One day this week, ideally tomorrow, please pay special attention to the locations you visit (e.g., bus stop, supermarket, school hall, etc.). When you log in to the study next week, you will be asked to list three places you spent time in on that day. If you want to make a note of the three places in order to help you remember them then please feel free to do so. Do not worry about recording whom you are with or the emotions you are experiencing when in the locations, only details of the places (i.e., the facts) are important. |
| Control Activity: Room Description | Please take a moment to think about your bedroom. Now, for the next 10 minutes, please write a detailed description of that room. Be as detail-oriented as possible, but try to leave out emotions, feelings, or opinions relating to the room. In other words, just describe exactly what is in the room. There is no need to worry about perfect grammar or spelling. Please be assured that anything you write will remain strictly confidential. |
| Positive Activity: Kindness | From time to time, we all perform acts of kindness for others. These acts may be large (like taking old clothes to a charity shop) or small (like opening a heavy door for someone), and the person for whom the act is done may or may not be aware of the act. Examples of kind acts include helping your parents cook dinner, doing a chore for your sister or brother, helping a friend with homework, or visiting an elderly relative.  One day this week, ideally tomorrow, you are to perform three acts of kindness—all three in one day. The three acts do not need to be for the same person, and it doesn’t matter if the person knows whether you did it or not. The three kind acts may be similar to the examples above, but they don’t have to be. When you log in next weekend, you will be asked to provide a brief description of each act you performed. If you want to make a note of what acts you did in order to help you remember them then please feel free to do so. Please do not do any kind acts that may place you or others in danger. |
| Positive Activity: Gratitude | Please take a moment to think back over your life and remember an instance when someone did something for you for which you are extremely grateful. For example, think of someone in your family who has been especially kind to you but may have never heard you express your gratitude. Now, for the next 10 minutes, write a letter of thanks to one of these individuals. There is no need to worry about perfect grammar or spelling. Please be assured that anything you write will remain strictly confidential.  Use the instructions below to help guide you through this process.   1. Use whatever letter format you like, but remember to write as though you are directly addressing the individual to whom you are grateful. If it is helpful, feel free to head the letter ‘Dear so-and-so,’ and end with ‘Sincerely, xxx.’ 2. Describe in specific terms why you are grateful to this individual and how his or her behavior affected your life. 3. Describe what you are doing now and how you often remember their efforts. 4. Although you may show or give this letter to anyone you please, we do not encourage you to do so. For the purpose of this study, the letter you write is an opportunity for you to express your gratitude freely without anyone else needing to see it. |
